# Compensatory mechanisms from different exercise intensities in type 2 diabetes: a secondary analysis of a 1-year randomized controlled trial

**DOI:** 10.1007/s00592-023-02038-7

**Published:** 2023-02-02

**Authors:** Inês R. Correia, Megan Hetherington-Rauth, João P. Magalhães, Pedro B. Júdice, Gil B. Rosa, Duarte Henriques-Neto, Asier Manas, Ignacio Ara, Analiza M. Silva, Luís B. Sardinha

**Affiliations:** 1 Faculdade de Motricidade Humana, Exercise and Health Laboratory, CIPER, Universidade de Lisboa Estrada da Costa, 1499-002 Cruz-Quebrada, Portugal; 2 CIDEFES - Centro de Investigação Em Desporto, Educação Física E Exercício E Saúde, Universidade Lusófona, Lisbon, Portugal; 3 GENUD Toledo Research Group, University of Castilla-La Mancha, Toledo, Spain; 4 CIBER of Frailty and Healthy Aging (CIBERFES), Madrid, Spain

**Keywords:** Compensation, HIIT, Spontaneous activity, NEPA, NEAT, Diabetes mellitus

## Abstract

**Aims:**

This investigation aimed to determine the effect of different intensities of training on non-exercise physical activity (NEPA) and estimated thermogenesis (NEAT) from a 1-year exercise randomized controlled trial (RCT) in individuals with type 2 diabetes mellitus (T2DM) on non-training days. Additionally, changes in NEPA and estimated NEAT in those who failed (low-responders) or succeeded (high-responders) in attaining exercise-derived clinically meaningful reductions in body weight (BW) and fat mass (FM) (i.e., 6% for FM and 3% for BW) was assessed.

**Methods:**

Individuals with T2DM (*n* = 80) were enrolled in a RCT with three groups: resistance training combined with moderate-intensity continuous training (MICT) or high-intensity interval training (HIIT) and a control group. Of the 80 participants, 56 (completed data) were considered for this secondary analysis. NEPA and estimated NEAT were obtained by accelerometry and body composition through dual-energy X-ray absorptiometry.

**Results:**

After adjustments, no time*group interactions were found for estimated NEAT in the MICT (β = − 5.33, *p* = 0.366) and HIIT (β = − 5.70, *p* = 0.283), as well as for NEPA in the MICT (β = − 452.83, *p* = 0.833) and HIIT (β = − 2770.76, *p* = 0.201), when compared to controls. No compensatory changes in NEPA and estimated NEAT were observed when considering both low-responders and high-responders to FM and BW when compared to controls.

**Conclusions:**

Both MICT and HIIT did not result in any compensatory changes in estimated NEAT and NEPA with the intervention on non-training days. Moreover, no changes in estimated NEAT and NEPA were found when categorizing our participants as low-responders and high-responders to FM and BW when compared to controls.

Trial registration clinicaltrials.gov ID.

NCT03144505.

## Introduction

Obesity is an underlying risk factor for type 2 diabetes (T2DM), in which exercise alongside with medication and nutrition are the most used strategies to prevent, control, and treat this condition [1]. Nonetheless, those undergoing exercise interventions may be subject to behavioral and metabolic adaptations in order to save energy, commonly described as the “compensatory effect of exercise” [2]. Almost 30% of exercise-induced increments in energy expenditure (EE) are compensated by physiological and behavioral adaptations such as a reduction in the resting metabolic rate, an increase in energy intake, and/or a reduction in physical activity levels [3, 4].

An important aspect of energy balance to consider for obesity management [5] is that of non-exercise physical activity (NEPA), which includes all the activities that do not pertain to volitional exercise, such as standing, household chores, or ambulation [6], and non-exercise activity thermogenesis (NEAT), which encompasses the EE associated with these activities [6]. NEAT is the most variable component of daily total EE, accounting for 15% to 50% of the total daily EE in highly sedentary and very active individuals, respectively [7–10]. Thus, compensation in both behavioral and physiological dimensions (i.e., NEPA and NEAT, respectively) could represent a physiological barrier for individuals to improve body composition and, thus, may be a source of inter-individual variability in the response to exercise [11]. This is of particular interest for those with T2DM, since maintaining weight loss is an important clinical goal with implications on glycemic control and cardiovascular risk [12].

Characteristics of the exercise dose may influence the degree and amount of change observed in NEPA and NEAT following an exercise intervention, with most of the information on this topic deriving from either healthy adults or overweight/obese individuals [13], with no interventions performed in T2DM. Moreover, these investigations did not distinguish exercise days from non-exercise days, where expected differences in NEPA and NEAT may occur [4, 14–17]. Therefore, this investigation will overcome the referred shortcomings by analyzing whether: (1) exercise intensity influences compensations in NEPA and estimated NEAT on non-exercise days following a 1-year exercise intervention; and (2) individuals who failed (low-responders or succeeded (high-responders) to attain exercise-derived clinically meaningful reductions in body weight (BW) and fat mass (FM) changed their NEPA and estimated NEAT.

## Methods

### Subjects and study design

**Fig. 1:**
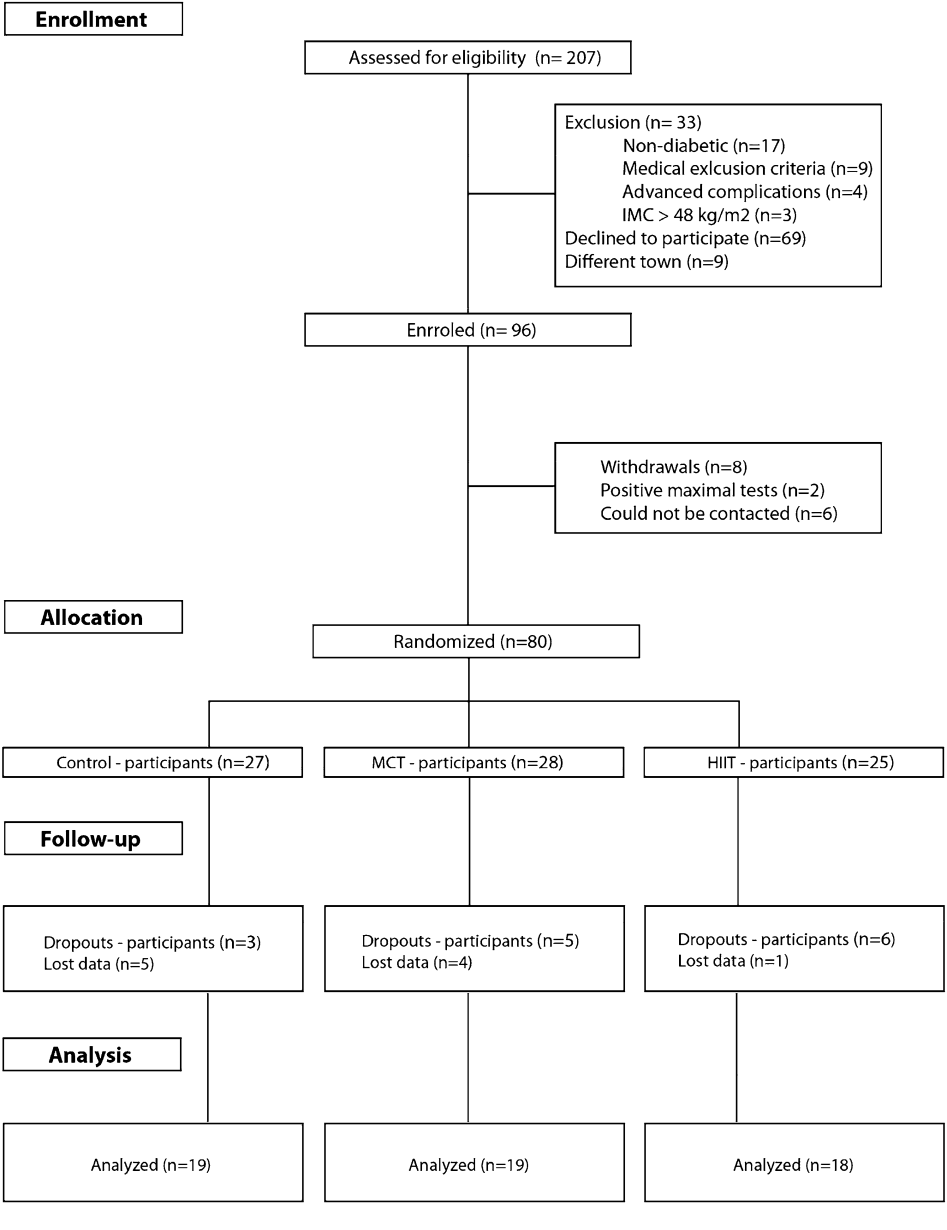
Study flowchart

This investigation is a secondary analysis of a 1-year randomized crossover trial conducted in individuals with T2DM that aimed to compare the effect of different exercise intensities on glycated hemoglobin as the main outcome. A total of 80 participants with T2DM completed baseline assessments and were allocated to one of three arms: (1) high-intensity interval cycling combined with resistance training (HIIT); (2) moderate-intensity cycling combined with resistance training (MICT); (3) control group. Sample size calculation procedures were based on a predicted glycated hemoglobin difference of 0.66 units, with a standard deviation of 1.2 units (α, 0.05; 1 − β, 0.80) and an expected dropout rate of 10% (Fig. 1) [18]. For this secondary analysis, and due to dropout and loss of data, the final sample consisted of 56 participants.

### Intervention protocol

The detailed protocol is described elsewhere [19]. Briefly, the participants performed three supervised and individualized sessions per week with both exercise groups having matched EE. The MICT group performed continuous cycling at 40 to 60% of the heart rate reserve (HRR). The HIIT group performed cycling with a 1:1 exercise to rest ration at 90% of the HRR followed by 1 min at 40–60% of the HRR. After the aerobic component, both groups performed a whole-body resistance training including one set of 10–12 repetitions of seated row, pull down, chest press, shoulder press, leg press, one leg lunge, dead bug, and regular plank. The control group was invited to an orientation session, where detailed exercise information was provided. Additionally, every 4 weeks, participants in all groups attended thematic sessions about important topics related with T2DM management. For all intervention groups, adherence to the exercise program was recorded and reported as a percentage of the total number of exercise sessions that were attended by each participant.

### Anthropometry and body composition

Participants’ weight and height were measured to the nearest 0.01 kg and 0.1 cm, respectively, on an electronic scale with stadiometer (Seca, Hamburg, Germany) according to the standardized procedures [20]. A whole-body dual-energy X-ray absorptiometry (Hologic Explorer-W, Waltham, USA) was used to estimate total body fat.

### Sensor-based data

Participants were instructed to wear an accelerometer (ActiGraph, GT3X model, Fort Walton Beach, FL) on the right hip for 7 days at baseline, 6-, and 12-months (during the exercise intervention). Data were recorded at a 100 Hz frequency, and downloaded into 10 s epochs. Troiano et al. cut-points and validation criteria were used to analyze the data [21]. An often overlooked issue in the literature concerns the use of accelerometers to determine NEAT and NEPA during an exercise intervention involving cycling and resistance training as the main stimulus. This issue has potential implications for the NEPA and NEAT calculations given that non-ambulatory activities are typically misclassified and not accounted for by the accelerometer [22]. Since these exercises were chosen during the D2FIT intervention, we considered using only the non-exercise days to avoid any biased outcomes. Moreover, the non-exercise days account for most of the days during the 7-day collection period.

#### Non-exercise activity thermogenesis determination

The ActiLife software and the refined 2-regression model Crouter equation [23] were used to determine the EE in METS, where the value of 1 MET was subtracted from the METS recorded for each hour on the non-exercise days to estimate the NEAT, expressed as kcals. The resulting kcals for each hour on the non-exercise days were summed and then averaged to estimate the average daily NEAT for valid days. The refined 2-regression Crouter equation has been shown to produce a better estimation of free-living EE compared to the single regression model approaches both in adults [23] as well as in older adults [24].

#### Non-exercise physical activity determination

All the activity counts were summed for each of the non-exercise days and then averaged to get the average NEPA per non-exercise day for each participant.

### Identifying individual exercise fat mass responders

Currently, there are no accepted guidelines for the percent of FM loss considered to be clinically meaningful. Therefore, we considered someone who had a FM loss greater than the typical error (TE) as clinically meaningful. The TE was calculated from the standard deviation (SD) of the differences in FM over 1-year in the control group, an approach suggested by Bonafiglia et al. [25] and used by others [26, 27]. The TE represents the technical error of measurement as well as the within-subject variability caused by changes in behavioral/environmental factors across an intervention. The TE for FM in our study was 1.87 kg. Hence, any individual with a FM loss > 1.87 kg (i.e., -6% FM from baseline) was considered to be a high responder, and individuals with FM $\le$ 1.87 kg were considered low responders. The same procedure was used to classify responders based on BW loss. The TE for BW was 2.64 kg. Any individual with a BW loss > 2.64 (i.e., -3% BW from baseline) was considered to be a high responder and individuals with BW loss $\le$ 2.64 kg were considered low responders.

### Statistical analysis

Descriptive statistics, including measures of central tendency (mean) and variability (standard deviation), were used to describe baseline characteristics of the control group, MICT, and HIIT. A one-way ANOVA with a Bonferroni adjustment for multiple comparisons was used to test differences in descriptive characteristics between the three groups and a chi-square test was used to assess differences in gender among the groups.

Generalized estimating equations were used to assess group by time interactions in NEPA and estimated NEAT on non-exercise days between the controls and exercise groups at 6-months and 1-year while using sex, age, wear time, and total number of trainings as covariates. Baseline NEPA and estimated NEAT were also included in their respective models as covariates given the significant difference between the groups at baseline. A least significant difference post hoc test was used to estimate the between and within-group effects on NEPA and estimated NEAT. A linear distribution for the response was assumed and an autoregressive correlation matrix was set to the data. Similarly, generalized estimating equations were used to assess group by time interactions in NEPA and estimated NEAT between the controls, low responders, and high responders for both FM and BW loss at 6 months and 1-year, while adjusting for sex, age, total number of trainings, wear time, and baseline NEPA or estimated NEAT.

A *p*-value of < 0.05 was considered statistically significant. Data analyses were performed using IBM SPSS Statistics version 22.0 (SPSS Inc., an IBM Company, Chicago, Illinois, USA).

## Results

The baseline characteristics are shown in Table 1. Data derived from accelerometry are for non-exercise days only. Overall, there were no differences between groups at baseline, except for NEPA and estimated NEAT, where the MICT group had higher values than the controls. On average, the adherence rate to both exercise protocols was 76.8 ± 21.2%

**Table 1:** Baseline characteristics of the participants by group

	Control (n = 19)	MICT (n = 19)	HITT (n = 18)	All sample (n = 56)
	Mean ± SD	Mean ± SD	Mean ± SD	Mean ± SD
Age (years)	60.7 ± 7.7	59.5 ± 6.4	56.0 ± 8.8	58.8 ± 7.9
Body mass (kg)	84.3 ± 15.8	83.0 ± 14.8	80.0 ± 16.5	82.5 ± 15.5
Height (cm)	163.7 ± 9.6	163.6 ± 7.9	165.6 ± 8.1	164.3 ± 8.6
BMI (kg/m^2^)	31.8 ± 4.6	31.6 ± 5.7	29.5 ± 5.3	31.0 ± 5.3
T2DM duration (years)	5.9 ± 5.6	8.7 ± 5.6	6.0 ± 3.9	6.9 ± 5.2
Fasting glucose (mg/dl)	142.0 ± 29.7	169.3 ± 75.1	161.6 ± 58.6	157.6 ± 57.7
HOMA2_IR	2.4 ± 1.5	1.9 ± 1.2	2.0 ± 1.2	2.1 ± 1.3
Adherence (%)	N/A	78.0 ± 18.40	75.6 ± 23.9	76.8 ± 21.2
Woman, n (%)	10 (53%)	10 (53%)	6 (33%)	26 (46%)
Whole-body fat (kg)	30.1 ± 6.3	30 ± 7.9	26.5 ± 10.2	28.9 ± 8.3
Sedentary Behavior (min/day)	647.6 ± 61.1	628 ± 107.6	665.7 ± 83.7	646.8 ± 86.1
LIPA (min/day)	178.9 ± 58.3	206.3 ± 74.7	176.5 ± 41.9	187.4 ± 60.6
Moderate PA (min/day)	25.8 ± 16.4	39.8 ± 24.6	40.9 ± 25.8	35.4 ± 23.3
Vigorous PA (min/day)	0.3 ± 0.4	0.5 ± 0.7	0.9 ± 1.4	0.6 ± 1.0
NEAT (kcal)*	543 ± 172.3	737.7 ± 283.9	629.7 ± 210.6	636.9 ± 237.4
NEPA (total counts)*	188,426.6 ± 74,006.9	267,821.4 ± 115,130.1	254,100.1 ± 108,589.6	236,473.5 ± 104,946.7
HbA1c (%)	6.9 ± 1.1	7.2 ± 1.6	6.9 ± 1.0	7 ± 1.2

*BMI* body mass index, *T2DM* type 2 diabetes mellitus, *MICT* moderate-intensity continuous training, *HIIT* high-intensity interval training, *HOMA2_IR* Homeostasis Model Assessment Insulin Resistance, *SD* standard deviation; *n* number of participants, *LIPA* light-intensity physical activity, *PA* physical activity, *HbA1c* glycated hemoglobin, *NEAT* non-exercise activity thermogenesis, *NEPA* non-exercise physical activity

^*^Differences between MICT group and control group at baseline *p* < 0.05 (α = 5%); a one-way ANOVA with a Bonferroni adjustment for multiple comparisons and chi-square test were used

**Fig. 2:**
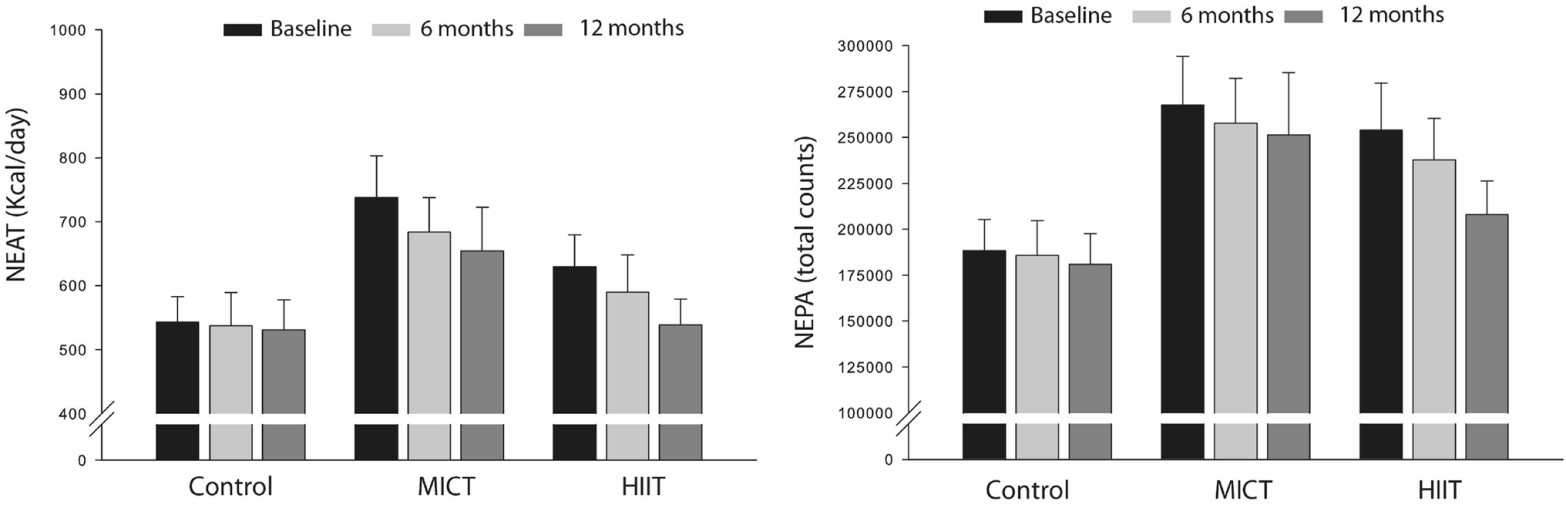
Estimated non-exercise activity thermogenesis (NEAT) and non-exercise physical activity (NEPA) measured at baseline, 6- and 12-months for all groups (control, MICT, and HIIT)

Behavioral (NEPA) and physiological (estimated NEAT) changes between the exercise groups and controls during the intervention period as well as between low responders and high responders for BW and FM loss are described in Table 2. Regardless of the exercise intensity (i.e., MICT or HIIT), there were no group by time interaction differences in NEPA and estimated NEAT over the 1-year exercise intervention compared to the controls after adjusting for sex, age, number of trainings, wear time, and baseline NEPA or estimated NEAT (*p* > 0.05). NEPA and estimated NEAT values for baseline, 6-, and 12-months are presented in Fig. 2. Although no statistical significance was reached (*p* > 0.05), this figure shows a trend toward decreasing NEPA and estimated NEAT overtime on the non-exercise days in the exercise groups, particularly those performing HIIT.

**Table 2:** Within- and between-group changes at baseline and after 1-year, in non-exercise activity thermogenesis and non-exercise physical activity for the control group and exercise groups (i.e., MICT and HIIT), as well as for the low responders and high responders based on a fat mass or body weight loss $\le 1.87\mathrm{ kg or }2.64\mathrm{ kg},\mathrm{ respectively}$. Betas are presented as unstandardized coefficients adjusted for sex, age, number of trainings, wear time, and baseline NEPA OR NEAT depending on the dependent variable, with respective 95% confidence intervals

Outcome	Control (n = 19)		MICT (n = 19)		HIIT (n = 18)		MICT*Control	HIIT*Control	MICT*HIIT
	Baseline	1-year	Baseline	1-year	Baseline	1-year	β (95% CI)	β (95% CI)	β (95% CI)
NEAT (kcal/day)	543.02 ± 172.27	530.91 ± 202.71	737.72 ± 283.87	654.57 ± 296.14	629.69 ± 210.58	538.15 ± 173.59	− 5.33 (− 16.88; 6.23)	− 5.70 (− 16.11; 4.71)	0.37 (− 11.28; 12.01)
NEPA (counts/min)	188,426.63 ± 74,006.93	180,957.97 ± 72,576.2	267,821.42 ± 115,130.15	251,417.67 ± 148,041.09	254,100.14 ± 108,589.62	207,923.03 ± 77,578.30	− 452.83 (− 4650.68; 3745.03)	− 2770.76 (− 7019.85; 1478.32)	2317.94 (− 2625.04; 7260.91)

Outcome	Control (n = 19)		FM LR (n = 24)		FM HR (n = 13)		FM LR * Control	FM HR * Control	FM HR * FM LR
	Baseline	1-year	Baseline	1-year	Baseline	1-year	β (95% CI)	β (95% CI)	β (95% CI)
NEAT (kcal/day)	543.03 ± 172.27	530.91 ± 202.71	640.47 ± 232.15	533.27 ± 182.24*	737.76 ± 274.00	674.01 ± 296.13	− 6.59 (− 16.71, 3.53)	− 4.26 (− 16.31, 7.80)	− 2.34 (− 14.23, 9.56)
NEPA (counts/min)	188,426.63 ± 74,006.93	180,958.00 ± 72,576.21	239,999.15 ± 101,365.32	192,746.62 ± 102,958.66*	286,025.10 ± 118,874.53	274,389.28 ± 125,406.81	− 2656.08 (− 6334.93, 1022.76)	− 323.23 (− 5212.39, 4565.93)	− 2332.86 (− 7490.35, 2824.63)

Outcome	Control (n = 19)		BW LR (n = 31)		BW HR (n = 6)		BW LR * Control	BW HR * Control	BW HR * BW LR
	Baseline	1-year	Baseline	1-year	Baseline	1-year	β (95% CI)	β (95% CI)	β (95% CI)
NEAT (kcal/day)	543.03 ± 172.27	530.91 ± 202.71	660.92 ± 225.21	560.29 ± 199.12*	750.63 ± 322.28	699.57 ± 340.54	− 6.42 (− 15.96, 3.12)	− 3.04 (− 18.33, 12.25)	− 3.39 (− 18.08, 11.31)
NEPA (counts/min)	188,426.63 ± 74,006.93	180,958.00 ± 72,576.21	251,959.32 ± 95,274.99	205,824.96 ± 99,878.43*	285,950.80 ± 147,703.34	296,227.62 ± 147,302.96	− 2748.10 (− 5980.79, 484.60)	1582.18 (− 5545.79, 8710.16)	− 4330.28 (− 11,426.48, 2765.93)

Coefficients are presented as unstandardized coefficients with the respective 95% confidence intervals

*NEAT* non-exercise activity thermogenesis, *NEPA* non-exercise physical activity, *MICT* moderate-intensity continuous training, *HIIT* high-intensity interval training, *SD* standard deviation, *n* number of participants, *FM* fat mass, *BW* body weight, *LW* low-responders; *HR* high-responders

^†*^Between-group changes significant at *p* < 0.05; * Within-group changes significant at *p* < 0.05 (α = 5%); Generalized estimating equations were used

As shown in Table 2, both FM and BW low-responders had no differences in changes in NEPA (FM: β = − 2656.08; − 6334.93, 1022.76; BW: β = − 2748.10; − 5980.79, 484.60) and estimated NEAT (FM: β = − 6.59; − 16.71, 3.53; BW: β = − 6.42; − 15.96, 3.12) when compared to the controls. Similar results were observed for high-responders, with no differences observed for both NEPA (FM: β = − 323.23; − 5212.39, 4565.93; BW: β = 1582.18; − 5545.79, 8710.16) and estimated NEAT (FM: β = − 4.26; − 16.31, 7.80; BW: β = − 3.04; − 18.33, 12.25) when compared to controls. Additionally, there were no differences in changes in NEPA and estimated NEAT between low- and high-responders following 1-year of exercise (*p* > 0.05). However, we observed a time effect for both FM and BW low-responders with a decrease in estimated NEAT (− 133.39 kcal (*p* = 0.031) and − 100.60 kcal (*p* = 0.026), respectively) and NEPA (− 54,597.40 counts/d (*p* = 0.021) and − 41,409.80 (*p* = 0.006) counts/d, respectively) following 1-year of exercise intervention.

## Discussion

To the best of our knowledge, this is the first experimental investigation that examined how estimated NEAT and NEPA are affected by a 1-year exercise intervention performed at different intensities in T2DM. Our results showed no compensatory decreases in NEPA and estimated NEAT on the non-exercise days following 1-year of exercise training, regardless of training protocol (HIIT/MICT). Moreover, whether an individual attained exercise-derived clinically meaningful reductions in BW or FM did not influence changes in NEPA and NEAT after 1-year of intervention.

Although it can be speculated that higher exercise intensities would promote more noticeable behavioral and physiological changes toward reducing NEAT and NEPA, the available evidence regarding this issue is still scarce, with none of the literature addressing the effect of exercise intensity on these compensatory mechanisms in the T2DM population. Indeed, the intensity issue has been previously reviewed by Washburn et al. in a population of healthy/overweight/obese adults, where no evidence was found for the intensity of the exercise influencing NEPA and NEAT following short to medium-term exercise interventions [28]. Our investigation corroborates and expands the previous findings, showing that regardless of the exercise intensity, while using exercise groups with matched EE, no compensations in both behavioral and physiological dimensions were observed in individuals with T2DM following a long-term 1-year intervention. However, beyond the scope of intensity, there have been conflicting and inconclusive results regarding the influence of higher volumes of exercise and compensatory mechanisms in NEPA [4, 14–17]. For instance, Schutz et al. reported that normal- and overweight women who achieved higher doses of exercise (60 and 90 min/day) following an 8-week intervention had higher reductions in NEPA compared to those with lower volumes (30 min/day) [14]. Nonetheless, other investigations found no NEPA/NEAT compensations after an exercise intervention, regardless of the exercise volume in healthy/overweight/obese adults [4, 16, 17]. In our investigation, the exercise volume was defined as 10 kcal/kg per week for both exercise groups, which was meant to match the physical activity guidelines [29], however, this may not have been a sufficient volume to trigger any compensatory response in our sample.

Another possible explanation for the absence of compensatory mechanisms in estimated NEAT and NEPA may be partially related with the inclusion of both aerobic and resistance training in our protocol. Research has suggested that the compensatory reduction in NEPA is lower after resistance training compared to aerobic exercise [30]. A randomized study conducted in sedentary healthy men who performed 16-weeks of aerobic plus 16-weeks of resistance training (6-weeks washout) concluded that aerobic training did not result in any behavioral compensation, while resistance training increased NEPA, particularly on the non-training days [31]. Therefore, the resistance component of the present investigation may have mitigated the potential compensatory responses resulting from the aerobic stimulus in both groups.

During exercise programs, the achieved weight loss often does not match the expected weight reduction due to potential compensatory mechanisms toward energy saving, such as reductions in NEAT and NEPA that attenuate the overall impact of the EE derived from the exercise sessions. Indeed, these compensations may contribute to the inter-individual variability in weight loss achieved from an exercise intervention, where those having clinically meaningful weight losses (i.e., high responders), potentially having higher changes in NEAT and NEPA when compared to low responders in weight loss. A recent systematic review reported that the participants who lost the most weight were also the ones who compensated the most with decreases in NEAT [13], suggesting that the energy imbalance resulting in weight loss may lead to energy conservation. However, most of these observations were dependent on the type of intervention used to induce weight loss, with 63% reporting declines in NEAT/NEPA from diet-only, whereas only 27 and 23% observed these compensatory behaviors with combined diet/exercise and exercise-only, respectively. Therefore, it is plausible that diet-only interventions for weight loss may be more prone to promote decreases in NEAT and NEPA when compared to exercise-based interventions.

In our exercise-based intervention, we observed no compensatory reductions in NEPA and estimated NEAT, after 1-year of intervention, when categorizing participants with T2DM as low or high responders for FM or BW, when compared to non-exercising controls. Conversely, Herrmann et al., using a long-term exercise intervention in overweight and obese adults reported that men who were categorized as low weight loss responders (< 5% weight loss) decreased their NEPA and NEAT levels as well as increased their energy intake to a greater extent when compared with the high responder group (≥ 5%) [32]. The observed differences between our intervention and the trial carried out by Herrmann were possibly due to different exercise volumes used. As aforementioned, in our intervention, participants achieved a targeted exercise volume of 10 kcal/kg/week, which is considerably lower when compared to the intervention designed by Herrmann (400–600 kcal/session) and, therefore, not enough to induce compensatory mechanisms. Interventions with higher volumes of exercise are likely needed to trigger noticeable compensatory decreases in NEPA and NEAT. Likely, the lack of meaningful BW or FM loss in the participants classified as low responders may be due to an increase in energy intake (as observed by Herrmann et al.), however, we were unable to verify this hypothesis given the lack of control for energy intake in our investigation. Hence, given the conflicting results in exercise-only studies, it is clear that more exercise trials are warranted to clarify the impact of body composition changes following exercise on NEPA and NEAT.

This investigation is not without limitations. Since this investigation aimed to assess NEPA and NEAT in free-living conditions, and given the known limitations of gold standard methods, such as indirect calorimetry or doubly labeled water to assess these constructs in the field, we opted to use motion sensors, while acknowledging some validation issues for estimating EE [22]. Nevertheless, the use of accelerometry is a feasible approach to estimate EE for ambulatory activities [33, 34], which were the most prominent activities in patients with T2DM on the non-exercise days. We acknowledge that only assessing NEPA and estimated NEAT on non-exercise days is a limitation, as part of the potential reductions in NEPA and estimated NEAT may happen immediately after the exercise sessions. However, assessing only non-exercise days allowed us to avoid any bias in our results given the known limitations of the accelerometers to detect and measure activities such as cycling and resistance training during the training days. Another limitation lies in the fact that changes in NEPA and estimated NEAT were only assessed at 0, 6, and 12 months. It is possible that reductions in NEPA and/or NEAT could have been found during the first weeks of the intervention, since it is well recognized that, acutely, the feeling of fatigue may yield decreases in NEPA and NEAT during the initial period [30].

As a major strength, it is important to highlight that this is the first investigation to explore the impact of different exercise regimes and the inter-individual variability in body composition changes resulting from a 1-year exercise intervention on NEPA and estimated NEAT levels in individuals with T2DM on non-exercise days. This is relevant considering that most studies have reported the combined changes on both exercise and non-exercise days, making it impossible to understand what is happening with NEPA and estimated NEAT on the non-training days following exercise interventions. This issue is even more relevant when longer-term interventions are planned, where exercise attendance rates tend to decrease over time.

## Conclusions

In contrast with other interventions geared toward weight loss where behavioral and physiological changes in NEPA and NEAT, respectively, may be expected, a more conservative and ecological 1-year exercise intervention for individuals with T2DM had no impact on NEPA and estimated NEAT on the non-exercise days, regardless of the exercise intensity or having a clinically meaningful BW and FM loss. These results provide valuable information for those individuals with T2DM who aim to improve their body composition through an exercise intervention, where no physiological or behavioral barriers are expected to compromise their energy balance.

## Data Availability

The datasets used during the current intervention are available from the corresponding author on reasonable request.

## Abbreviations

BMI: Body mass index
BW: Body weight
EE: Energy expenditure
FM: Fat mass
HIIT: High-intensity interval training
HRR: Heart rate reserve
MET: Metabolic equivalents
MICT: Moderate-intensity-continuous training
NEAT: Non-exercise activity thermogenesis
NEPA: Non-exercise physical activity
SD: Standard error
T2DM: Type 2 diabetes mellitus
TE: Typical error
